# Analysis of Advanced Pore Morphology (APM) Foam Elements Using Compressive Testing and Time-Lapse Computed Microtomography

**DOI:** 10.3390/ma14195897

**Published:** 2021-10-08

**Authors:** Matej Borovinsek, Petr Koudelka, Jan Sleichrt, Michal Vopalensky, Ivana Kumpova, Matej Vesenjak, Daniel Kytyr

**Affiliations:** 1 Faculty of Mechanical Engineering, University of Maribor, Smetanova ulica 17, 2000 Maribor, Slovenia; matej.borovinsek@um.si (M.B.); matej.vesenjak@um.si (M.V.); 2 Institute of Theoretical and Applied Mechanics, Czech Academy of Sciences, Prosecka 809/76, 190 00 Prague, Czech Republic; koudelkap@itam.cas.cz (P.K.); sleichrt@itam.cas.cz (J.S.); vopalensky@itam.cas.cz (M.V.); kumpova@itam.cas.cz (I.K.)

**Keywords:** advanced pore morphology (APM) foam, computed microtomography, in-situ mechanical testing, compressive loading, deformation behaviour, porosity analysis

## Abstract

Advanced pore morphology (APM) foam elements are almost spherical foam elements with a solid outer shell and a porous internal structure mainly used in applications with compressive loading. To determine how the deformation of the internal structure and its changes during compression are related to its mechanical response, in-situ time-resolved X-ray computed microtomography experiments were performed, where the APM foam elements were 3D scanned during a loading procedure. Simultaneously applying mechanical loading and radiographical imaging enabled new insights into the deformation behaviour of the APM foam samples when the mechanical response was correlated with the internal deformation of the samples. It was found that the highest stiffness of the APM elements is reached before the appearance of the first shear band. After this point, the stiffness of the APM element reduces up to the point of the first self-contact between the internal pore walls, increasing the sample stiffness towards the densification region.

## 1. Introduction

**Figure 1:**
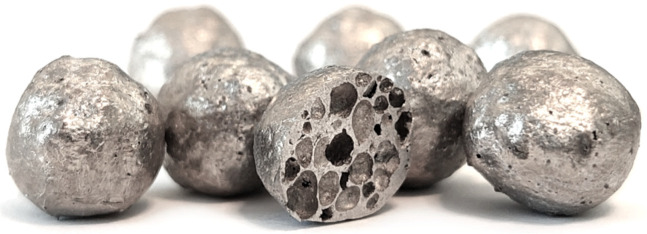
Advanced pore morphology foam elements.

Advanced pore morphology (APM) foam elements (Figure 1) are almost spherical elements made from closed-cell foam and enveloped with a solid outer shell [1,2,3], which were developed by the Fraunhofer Institute IFAM in Bremen [4,5]. They can be produced in different diameters from 3 to 15 mm with a bulk density between 500 and 1000 kg/m^3^ [4]. APM foam elements can be used as un-bonded or bonded hybrid cellular structures [4,5], where the bonding is usually performed using a polymer (e.g., Polyamide PA12, Araldite AT 1-1) [6,7,8]. When all the voids between the spherical APM elements are filled with a polymer filler, an APM-based polymer matrix foam is obtained with improved mechanical properties [9,10].

Due to their specific properties [11,12] and their versatile combinations with other filler materials, APM foam elements have a wide range of applications in layers of composite materials [13,14], in components for functional thermal conductivity [15], in components for absorbing impact energy [16] and vibrations [7] and inside hollow construction parts for their reinforcement against local wall buckling [5,17,18]. Using them as a filler material inside hollow components is especially advantageous, since they can easily fill even complex internal cavities of hollow components [19].

In general, APM foam elements have a characteristic stress–strain response in compression. Their mechanical properties have been analysed in detail in many studies using experimental testing and advanced computer simulations [5,6,11,12,15,20]. Their mechanical properties strongly depend on their internal foam geometry and morphology (e.g., the internal porosity distribution, pore distribution and pore sizes of APM foam elements). They were analysed in detail in [1,2,21], where X-ray computed microtomography (XCT) was used to inspect the internal structure of the undeformed and deformed APM foam elements, since XCT offers advantages in combination with both the volumetric approach and micron to sub-micron resolution even with the use of alaboratory equipment setup [22,23,24,25].

The existing studies focus primarily on the mechanical response or the internal structural changes in the APM foam elements, adding numerical simulations to better understand the mechanical behaviour of APM foam elements. Therefore, such studies give no direct answer to how the deformation of the internal structure and its changes are related to its mechanical response. With the advancements achieved in the field of porous solids for structural applications [26,27,28,29,30,31,32] and deformation energy absorption [33,34,35,36,37], advanced XCT imaging methodologies have been sought to reveal the deformation processes in the microstructure driving the overall mechanical response of the developed materials [38,39,40,41]. In particular, time-resolved XCT (4D XCT) experiments allowing for the characterisation of porous solids in response to mechanical loading has been extensively used, as it enables one to capture the deforming microstructure during the in-situ experiment performed in the XCT scanner either in discrete load steps or continuously throughout the loading procedure (so-called on-the-fly XCT) [42,43].

Numerous ex-situ compression experiments on metal foams [22,37,44] were already reported, including our previous study [1] on APM foam elements comprising ex-situ XCT imaging of samples compressed in eight loading steps. In-situ time-lapse measurements of metal foams in a limited number of loading cases can be found in the literature as well [45,46]. However, the new approach presented in this paper enables a direct comparison between the mechanical response and the internal structure of the foam and gives results on how the deformation of the internal structure affects the mechanical response of the APM elements in high detail and at least 25 loading steps up to 50% of overall compressive strain.

## 2. Methods

### 2.1. In-Situ Compressive Testing

**Figure 2:**
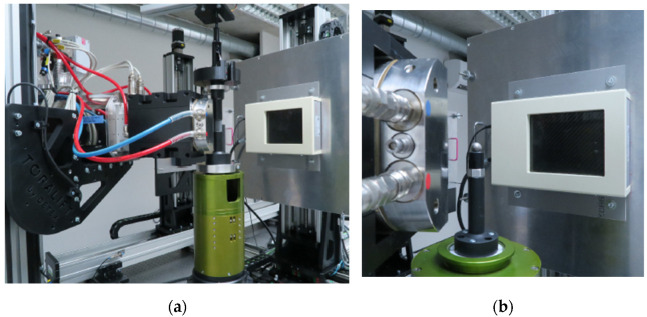
Experimental setup comprising the in-situ loading device mounted in the CT scanner: (**a**) An overview showing the X-ray source, the loading device on the rotary stage, and the detector; and (**b**) detail of the loading device with the demounted carbon-fibre loading chamber with an emplaced APM sample.

Mechanical experiments were performed using an in-house designed table-top loading device allowing in-situ 4D XCT experiments [47] to be performed with a modified loading chamber. To optimise the imaging procedure and obtain the best possible geometrical magnification of 9.85×, a 3-ply carbon fibre tube with a diameter of 20 mm and a loading capacity of 1000 N equipped with an LCM300 (Futek, Irvine, CA, USA) load-cell was used. Slip rings allowing the free rotation of the loading device in the XCT scanner were used to connect the device to the control unit. The device was mounted on the high-precision rotary stage of the XCT scanner (Figure 2a). Figure 2b depicts, in detail, the opened loading device with the placed APM sample. Because the device was operated close to its load-bearing limit, corrections to the acquired loading curves for the overall stiffness of the device were performed by the plate-to-plate calibration test, which resulted in 180 µm/kN of overall stiffness. For the same reason, a time-stability test of the device was performed by compressing the steel cylinder up to 1000 N, followed by a 10 min measurement phase. After 10 min, the force decreased to 976 N (2.5%) without a detectable encoder response proving that the time-stability of the measurement system guarantees high reliability and repeatability of the measurement. Thus, the force decreases in every load step are caused dominantly by the sample relaxation and not the loading device itself.

In this study, the APM foam elements produced from the AlSi7 alloy powder and foamed using TiH2 foaming agent were used. The foam elements were not coated with a bonding material and had a nominal diameter of 10 mm. More details about the manufacturing process can be found in [5,6]. Previous studies [1] showed that such foam elements contain a small number of large pores with a diameter of up to 3 mm and a large number of micropores with diameters as small as 4 μm.

A set of nine APM samples having a quasi-spherical shape with an outer diameter of 10.4 ± 0.4 mm, a meridian diameter of 10.1 ± 0.2 mm (measured by a mechanical calliper), and a weight of 387.2 ± 1.1 mg were subjected to uni-axial compression to investigate their deformation behaviour and microstructural characteristics. Three APM foam samples were compressed and scanned in-situ, while the rest of the samples were only investigated for their mechanical response. All the mechanical tests (i.e., both the preliminary tests and the time-lapse XCT imaging) were performed using a loading rate of 5 µm/s. During the radiographical imaging, XCT scans were performed in 200 µm displacement increments corresponding to approximately 2% of the overall compressive strain. The experiments were terminated by reaching approximately 50% strain corresponding to the end of the plateau region and the start of the densification.

The compressive response of the samples was measured using a load-cell signal with 0.1 N precision and a position encoder signal with 0.25 µm precision. A sensor readout rate of 100 Hz was selected and the experiments were controlled using in-house developed control software [48].

### 2.2. Computed Microtomography

For the radiographical imaging, an XWT-240-SE (X-RAY WorX, Garbsen, Germany) transmission-type X-ray tube operated at an acceleration voltage of 90 kV and a target current of 333 µA was used to irradiate the sample. A Dexela 1512 NDT (PerkinElmer, Waltham, MA, USA) flat panel X-ray detector based on a GOS scintillator and CMOS technology with an active area of 145.4 × 114.9 mm^2^ and a native resolution of 1944 × 1536 px was used for the image acquisition. Each tomographical scan of the time-lapse measurement consisted of 800 equiangular projections with a 90 ms acquisition time. The overall scanning time per one tomography was 6 min including the image acquisition time, time of detector readout, and rotary stage movement. In total, the in-situ experiments consisted of 26 or 27 scans per each sample. The reconstruction of the 3D images was performed using an FDK filtered backprojection reconstruction algorithm [49] implemented in VG Studio MAX 3.4 (Volume Graphics, Heidelberg, Germany). The resulting 3D images with a voxel size of 7.6 µm and dimensions of 1943 voxels × 1943 voxels × 1536 voxels were used in the further analyses after binarisation in MATLAB (MathWorks, Natick, MA, USA) using a global intensity threshold. This resolution allows to identify the pores with diameter bigger than 15 µm. In general the influence of micropores on deformation behaviour is negligible.

**Figure 3:**
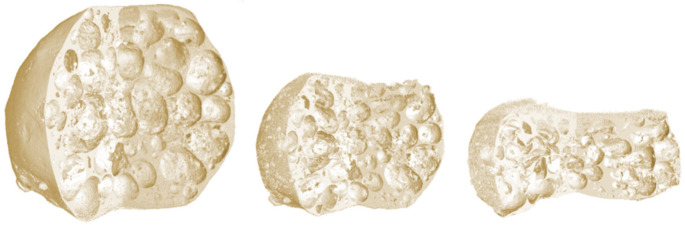
Visualisation of a deformed APM sphere—reconstructed 3D image captured after loading steps 1, 13 and 25.

The threshold value was determined based on a histogram evaluation in VG Studio MAX 3.4 by identification of a local minimum in the histogram of the reconstructed 3D image, since this minimum is located between the histogram peaks corresponding to the background intensities and APM material intensities and is, therefore, a good threshold value for binarisation. This enabled one to suppress the artifact of the cone-beam irradiated loading platens at the cost of loss of information near the loading platens (Figure 3), which was, however, outweighed by the substantial decrease in the noise in the microstructure of the sample. Additionally, special attention during the binarisation was paid to the conservation of the mass in the series of binarised 3D images. Thus, the threshold value was individually selected for each 3D image in such a way that the total volume of the APM base material of the binarised 3D image remained constant through all the deformation steps. The magnitude of error resulting from this procedure increases with the overall compression of the specimen as the sample height is lower and the influence of reflections due to the cone-beam geometry becomes more significant.

Nevertheless, due to the sufficient contrast to noise ratio in the reconstructed 3D images, it was possible to treat the photon scatter artefact in the vicinity of the loading platens during the binarisation procedure without further measures. However, an error resulting from such a binarisation procedure with a magnitude of few percent can still be expected.

**Figure 4:**
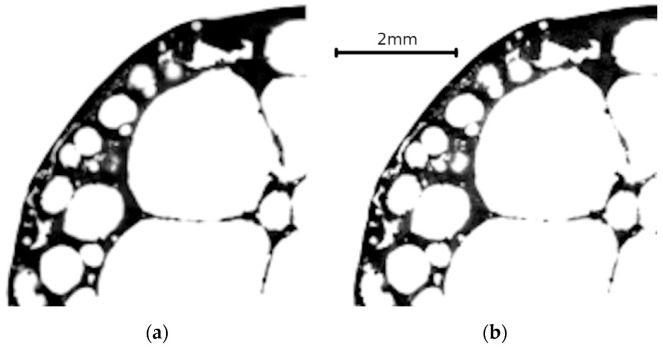
Undeformed APM sphere—transversal slices in the reconstructed 3D images of the high-quality reference CT scan (**a**) and the CT scan captured after the first loading step (**b**).

Furthermore, to quantify the effect of the relatively low number of projections (800) per tomography used in the time-lapse measurement for the scan speed increase, each sample was subjected to a high-quality reference CT scan with 2400 projections prior to the loading procedure. Figure 4 shows a comparison of transversal slices from the reconstructed 3D images of the high-quality CT scan and the first loading step of the time-lapse CT measurement.

Then, the reconstructed 3D images from this high-quality CT and undeformed state of the time-lapse measurement of every sample were compared. Here, the difference between the binarised 3D images was selected as a measure of conformity. Thus, the binarised 3D image of the reference scan was subtracted from the binarised 3D image of the zero-loading state and the total number of non-zero elements (i.e., voxels corresponding to the difference in the identified material of the sample) was calculated. From the three investigated samples, the average ratio of the dissimilar voxels to the total number of voxels in the 3D reconstructed images was 1.95%. In the worst case, the relative difference was 5.85%, which corresponds on average to 1.75 × 10^5^ dissimilar voxels in each of the 1943 slices in the reconstructed 3D image. Still, the majority was distributed around the photon scatter artefact caused by the aluminium loading platens.

### 2.3. Geometrical Analysis

**Figure 5:**
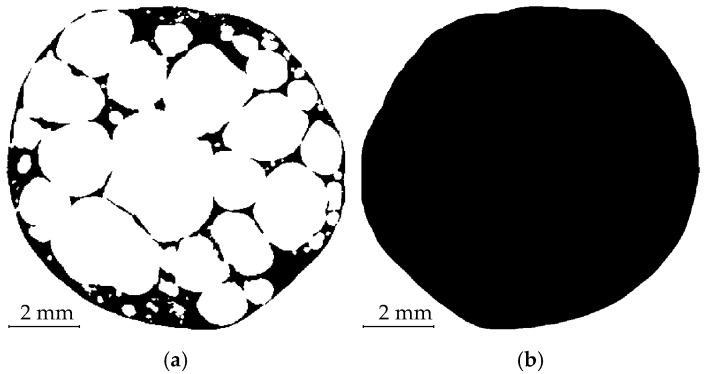
Binarised APM foam sample transversal slice with pores opened to the outside (**a**) and a filled APM foam cross-section using the developed filtering procedure (**b**).

To determine the changes in the APM foam elements’ outer shape, volume and porosity, the outer shape of the APM element had to be found for each 3D binarised image. In the case of porous materials, this is not a trivial task since some pores are opened towards the outside (Figure 5a) and no explicit border between the inside and outside of the foam exists. A new image filtering procedure to separate the inside of the APM foam from its outside was developed for that reason.

**Figure 6:**
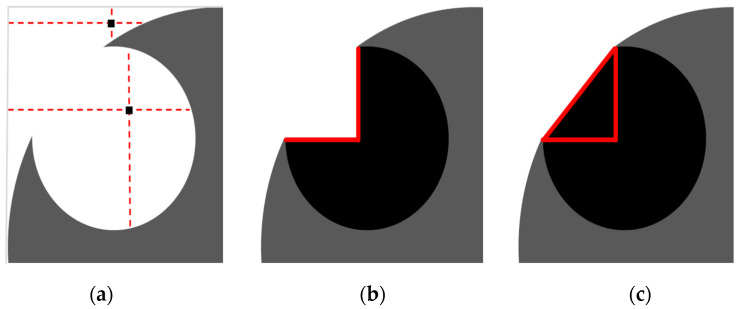
Image sequence of the proposed filtering procedure used to determine the outer shape of the APM foam elements.

The filter operates on one transversal slice at a time. For each pixel of the slice, four lines are projected to the image border in the following directions: left, right, up and down. Figure 6a shows a porous material (grey colour) with a pore opened to the outside (white colour) and two arbitrary pixels (black colour) with their projected lines (red colour). If at least two out of four lines reach the image border, the pixel is categorised as an outside pixel otherwise it is classified as an internal pixel. The result of this procedure is shown in Figure 6b, where all the newly found internal pixels are shown in black colour. The resulting material outer shape (grey and black colour) after this procedure has sharp corners and does not describe the outer shape appropriately. For this reason, an additional step was added to the filtering procedure. First, a border between the newly found internal pixels and the remaining outside pixels was determined (red colour in Figure 6b). The resulting border is comprised of two orthogonal line segments that separate the internal and external pixels. Secondly, the endpoints of the line segments were determined and used to construct a triangle, as shown in Figure 6c. Then, pixels inside the triangle are added to the newly found internal pixels and lastly, all the newly found internal pixels are merged to the pixels representing the porous material. In this way, a filled area of a single APM foam slice is determined (Figure 5b). Repeating this filtering procedure on all the recorded slices results in a filled APM foam volume separated from the background.

The filtering procedure enables one to determine the filled slice area $A_{f}$ and, assuming a circular shape of the filled area, a filled circle radius $r_{f}=\sqrt{A_{f}/\pi }$ was computed for each slice to observe the changes in the outer APM foam element shape during its deformation. Additionally, the area of the pores $A_{p}$ was determined to evaluate the slice porosity $p_{sc}=A_{p}/A_{f}$. Using the area values of all the slices combined and multiplying them with a voxel height of 7.6 µm, an average sample porosity was computed $p_{avg}=V_{p}/V_{f}$, where $V_{p}$ represents the volume of all the pores and $V_{f}$ represents the filled sample volume.

## 3. Results

### 3.1. In-Situ Compressive Test Results

**Figure 7:**
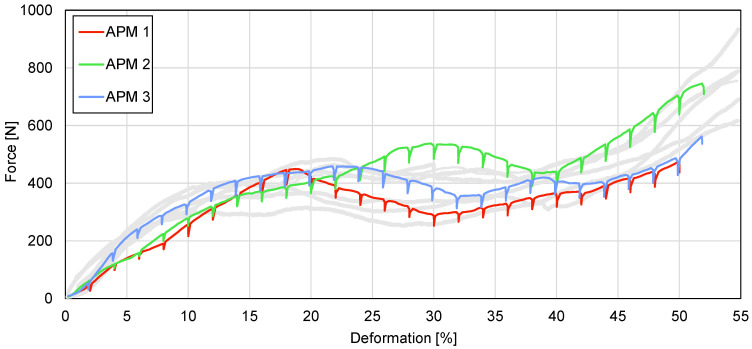
Loading curves of the preliminary tests (light-grey colour) and the three samples subjected to the time-lapse XCT imaging. The force decreases apparent in the curves were caused by the material relaxation during each CT scan.

The compressive behaviour of the samples is shown in the form of force–deformation curves in Figure 7. The stiffness-corrected loading curves obtained from the preliminary tests (grey lines in Figure 7) are in good agreement with the previously published results [12] and the trends of the deformation behaviour were not significantly affected by the intermittent loading during the time-lapse tomographical imaging (colour lines in Figure 7).

**Figure 8:**
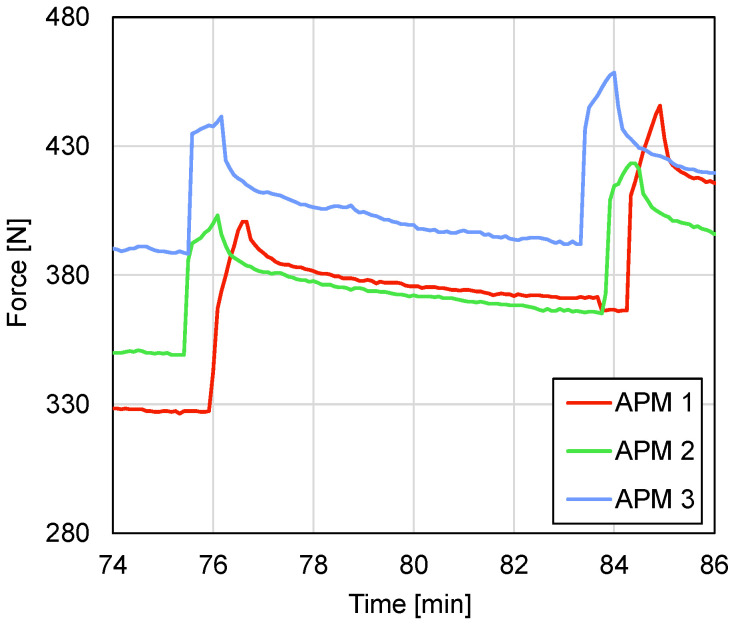
Time-dependent force decrease during one tomographical scan.

**Figure 9:**
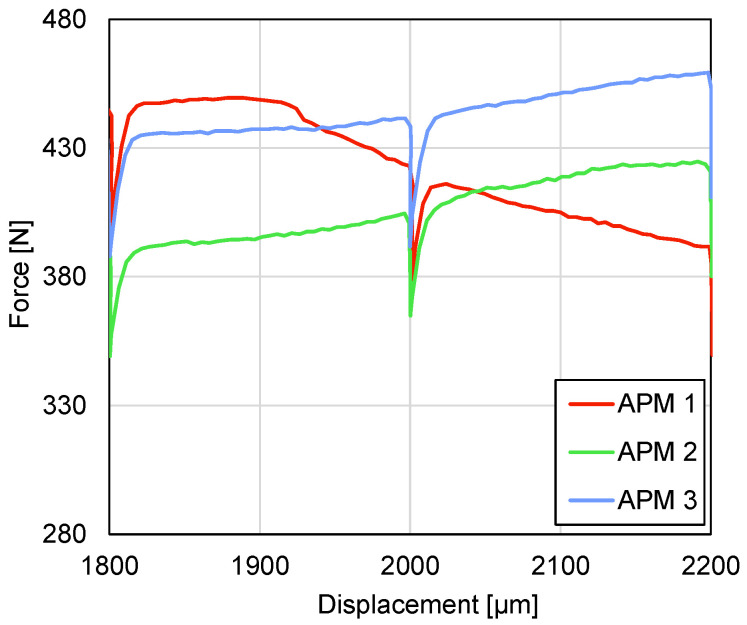
Force recovery in a single loading step.

During the intermittent in-situ loading procedure, the anticipated force decreases with a magnitude of 10–15% of the actual load occurred due to the release of elastic energy stored in the specimen and the loading device itself, causing the measured relaxation effect [50]. In every case, the major part of the decreases occurred in the first 2 min (Figure 8) after the interruption of the platen displacement. Therefore, a 2 min delay between the termination of a given loading step and the start of the tomographical scan was held. It can be seen that, in the next loading step, the force recovered back to the level of previous loading steps in 20–30 µm increment (Figure 9).

The compressive force–deformation curves have a characteristic shape for porous materials even though the samples were spherical and did not have a constant cross-sectional area. At the beginning of the loading procedure, a linear increase in the force up to the local maximum is followed by a decrease to a minimum plateau value. After that, the force increases in the transition to the densification region at deformations of approximately 50% of the overall compressive strain. The force values at the first force peak are equal to 449, 533 and 458 N at the deformations of 18.8%, 31.0% and 23.1% for APM foam samples 1, 2 and 3, respectively. The lowest plateau forces (neglecting the relaxation force drops) are equal to 292, 417 and 357 N at the deformations of 30.4%, 39.4% and 32.9% for APM foam samples 1, 2 and 3, respectively. The values for characteristic points in the force–deformation diagrams are summarised in Table 1

### 3.2. Micro-Computed Tomography Results

**Figure 10:**
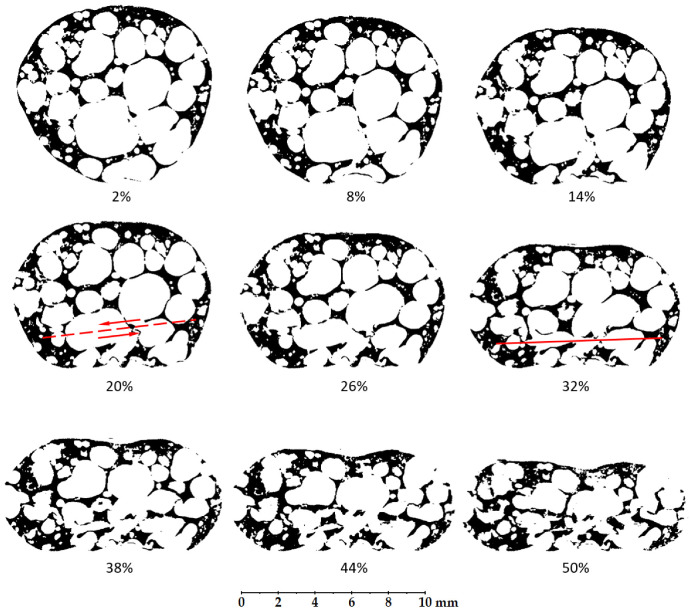
Lateral cross-sections of the APM foam sample 1 at deformation levels from 2% to 50%.

The deformation behaviour of the internal foam structure is shown in Figure 10, where the vertical cross-sections of APM foam sample 1 are shown at deformation levels from 2% to 50% using a step size of 6%. The images show that the deformation of the sample starts at the bottom support, where the bottom-most pore collapses first (from 2% to 14%). Then, a shear plane appears between the top and the bottom part of the sample around the deformation of 20%. The top part of the sample moves to the left while the bottom part is pushed to the right as shown by the arrows in Figure 10. After this transverse deformation of the sample, all the pores are compressed until the pores at the bottom collapse so much that their internal walls come into contact with each other at a deformation of about 30% (red line in Figure 10). As the deformation of the sample increases, the other pores are compressed until additional contact layers appear. The image of the last deformation stage shows that the sample retains a high porosity even at the final deformation of 50%. The other two APM foam samples have similar general behaviour but have different deformation levels at the appearance of the shear plane and first contact layer. The shear plane appears at the deformations of 30% and 22%, while the first contact layer appears at the deformations of 38% and 32% for APM foam samples 2 and 3, respectively.

### 3.3. Geometrical Analysis Results

**Figure 11:**
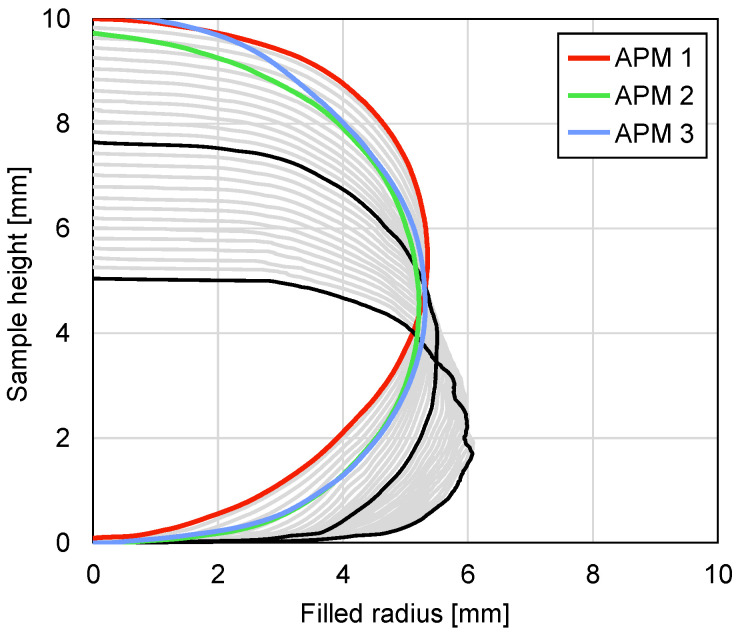
Filled radius of all the APM foam samples in colour and filled radius changes of the APM foam sample 1 in a grey colour with a deformation step of 2%; the black colour represents APM foam sample 1 radii at 26% and 50% deformation.

The deformation of the outer sample shape was analysed using the value of the filled circle radius $r_{f}$. Figure 11 shows the value of the filled radius on the transversal slice of all three APM foam samples in colours and the filled radius changes of the APM foam sample 1 in dependence on the sample height in a grey colour with a deformation step of 2%. The black lines represent the values of the filled radii at 26% and 50%. The results show that the undeformed shape of the samples is close to a spherical shape and that the undeformed sample heights are equal to 10.0, 9.73 and 10.1 mm, while the maximum undeformed filled radii are equal to 5.35, 5.22 and 5.31 mm for samples 1, 2 and 3, respectively.

With the increase in the deformation, the outer shape of APM sample 1 mostly compresses at the top and at the bottom of the sample, which is evident from the fact that the spherical sample shape is flattened at the bottom and the top of the sample. At the same time, the value of the maximum filled radius for each deformation stage increases from 5.35 to 5.51 mm and finally to 6.08 mm at the deformation stages of 0%, 26% and 50%, respectively.

**Figure 12:**
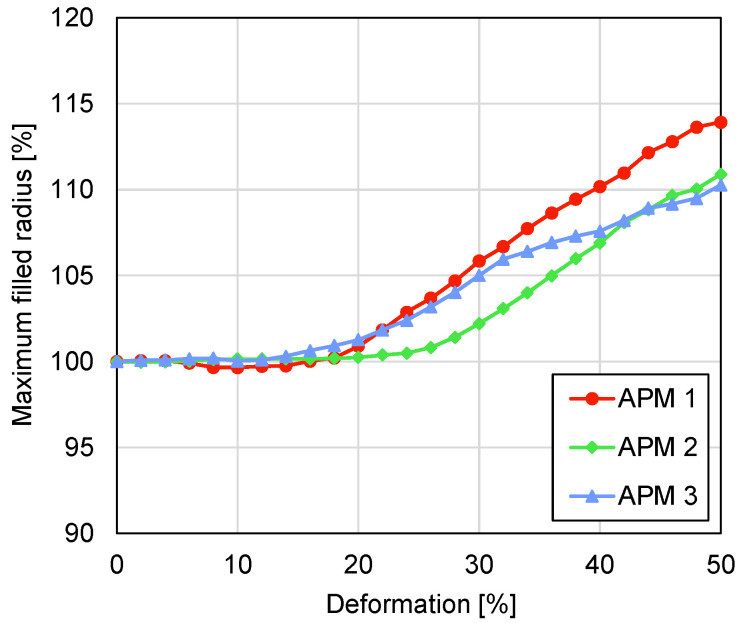
Maximum filled radius plotted against deformation.

The comparison of the outer shape changes between the different samples was carried out by comparing the maximum filled radius and the normalised filled volume. Figure 12 shows the value of the maximum filled radius of all three APM foam samples in dependence on the deformation. The maximum filled radius remains constant at the beginning of the sample deformation with a radius change below 1% up to the deformations of 22%, 28% and 20% for samples 1, 2 and 3, respectively. After this point, the filled radii increase monotonically up to the final values of 113%, 111% and 110%, respectively.

**Figure 13:**
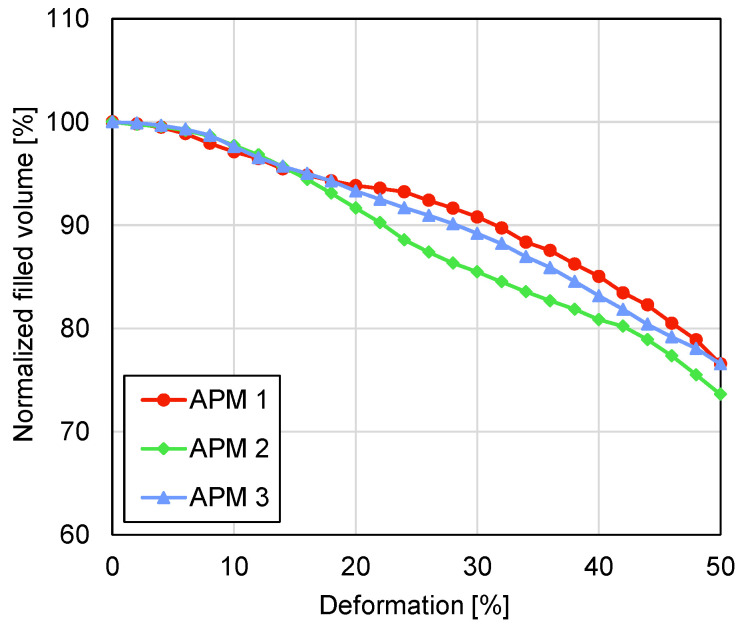
Normalised filled volume plotted against deformation.

The normalised filled volumes of the APM samples are shown in Figure 13 The volumes of all the samples monotonically decrease with an increase in the sample deformation. By observing the rate of the volume change per deformation step (curve inclination), a point, where the rate of volume change decreases to a local minimum, can be found in all three curves. That point occurs at the deformations of 22%, 30% and 18% for samples 1, 2 and 3, respectively. At the final deformation of 50%, the filled volumes equal 76.6%, 73.6% and 76.6% for APM foam samples 1, 2 and 3, respectively.

**Figure 14:**
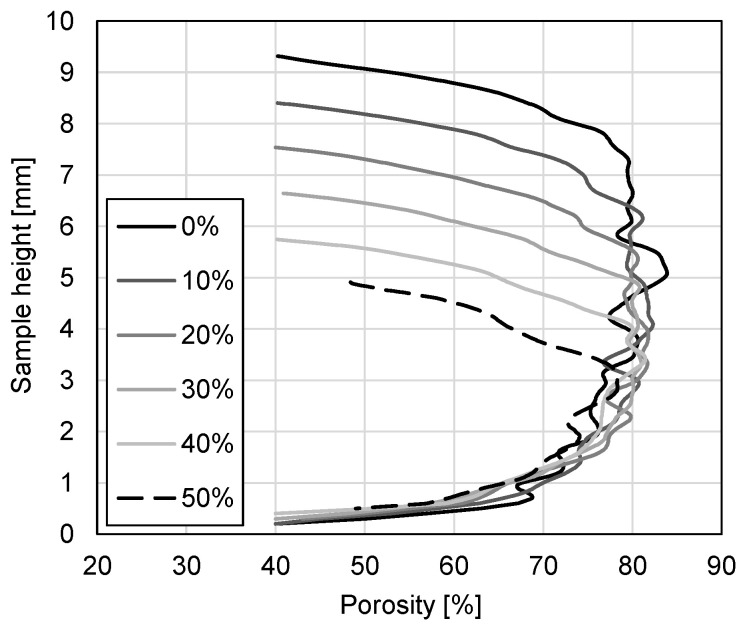
Cross-section porosity of APM foam sample 1 in dependence on the sample height and deformation.

The internal changes in the APM foam samples during the compressive deformation were examined by analysing the slice of the sample and average porosity. The slice porosity changes for APM foam sample 1 due to the applied deformation in dependence on the sample’s height are shown in Figure 14. The porosity distributions for all the deformation stages show an almost constant porosity in the middle of the sample that rapidly drops at the top and bottom of the sample. The porosity drop is the result of the APM foam element’s outer integral skin layer that is mainly solid (Figure 3) and represents a larger cross-section area at the top and bottom of the APM foam sample because of its spherical shape. The highest porosity for APM foam sample 1 was determined for an undeformed state at the height of 5.04 mm with a value of 83.8%. As the deformation increases, the porosity in the middle of the sample decreases from around 80% to 75% on average.

**Figure 15:**
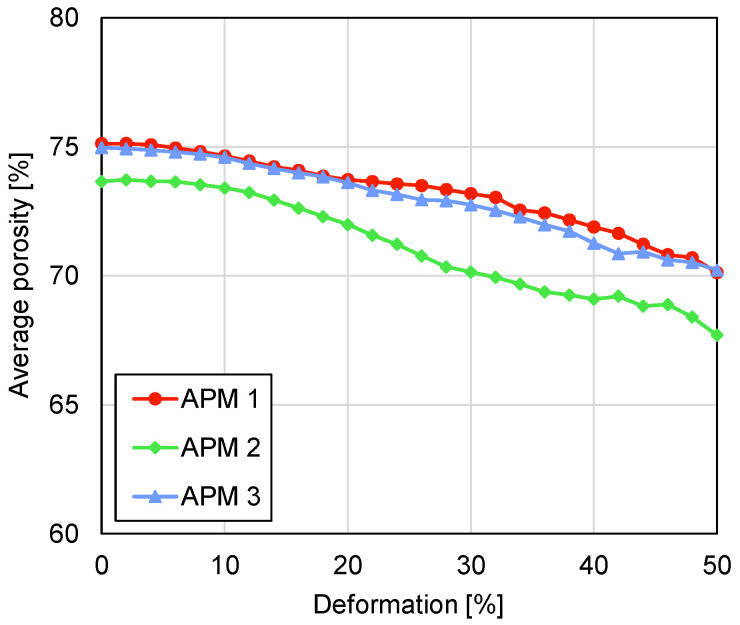
Average sample porosity plotted against deformation.

The decrease in the average sample porosity is also visible in Figure 15, which shows the average sample porosity $p_{avg}$ in dependence on the deformation. The APM foam samples average porosity before the deformation is equal to 75.1%, 73.6% and 75.0% for APM foam samples 1, 2 and 3, respectively. Assuming a sample’s ideal spherical shape, an averaged measured diameter of 10.1 mm, an averaged mass of 387.2 mg and an aluminium density of 2700 kg/m^3^, the computed average sample porosity is equal to 73.4% and is in good agreement with the porosity measured from the reconstructed 3D images. During the deformation of APM foam sample 1, its porosity decreased from 75.1% to 70.1%, it decreased from 73.6% to 67.7% for sample 2 and it decreased from 75.0% to 70.2% for sample 3. The porosity changes yield 5.0%, 5.9% and 4.8% for samples 1, 2 and 3, respectively. Observing the rate of porosity change per deformation step (curve inclination), a point can be found in all three curves when the rate of porosity change is decreased to a local minimum. That point occurs at the deformations of 20%, 30% and 26% for samples 1, 2 and 3, respectively.

## 4. Discussion

Using computed microtomography and in-situ compressive testing of APM foam samples enables the simultaneous evaluation of the mechanical and geometrical changes in samples. The overall shape of all three mechanical responses in Figure 7 is characteristic for porous materials, but reaches the plateau level at higher deformation levels in comparison with other porous materials [51]. The reason for such a response is the spherical shape of the APM foam samples. A spherical shape does not have a constant cross-sectional area in the direction normal to the applied compressive load. Thus, when the compressive load is applied, regions with a smaller cross-sectional area are deformed first, slowly transferring the deformation to regions with a larger cross-sectional area. Therefore, the mechanical response of spherically shaped samples is less stiff at the beginning when the top and the bottom regions of the sample are deformed, and the plateau region is reached at a higher deformation level. Figure 10, displaying a lateral cross-section of the deformed sample, confirms that the deformation of APM foam sample 1 started at the bottom region, i.e., where the cross-sectional area of the sample was the smallest. The top region clearly deformed later, at the deformation of about 20%. This is related to the fact that, at the top of the sample, there was more material resulting in a lower porosity and higher stiffness of the region. The lower porosity of the top region in comparison with the bottom region is also evident from Figure 14, where the porosity change rate (incline) towards the sample centre is smaller at the top, making the top region less porous and thus stiffer.

The compressive force response in Figure 7 shows the similar behaviour of APM foam samples 1 and 3 with a similar value of the first force peak, while sample 2 exhibits stiffer behaviour in the overall force response and reaches a higher first force peak value. The filled radius analysis shows that APM foam sample 2 is the smallest of the analysed samples and considering that the mass of the samples is almost equal, it follows that sample 2 has the lowest sample porosity. That conclusion is confirmed by the average porosity analysis shown in Figure 15, where sample 2 shows a porosity that is about 1.4% lower in comparison with samples 1 and 3.

Comparing the force response of APM foam sample 1 with its internal deformation shown in Figure 10 in terms of the deformation levels, it is evident that the first force peak is reached at the appearance of the shear plane. The first force peak was measured at the deformation of 18.8%, while the shear plane was observed at the deformation of 20%. At the appearance of the shear plane, the top and the bottom regions of the sample move transversally, since the shear plane is inclined towards the support plates, causing a decrease in the sample stiffness and, thus, a drop is recorded in the force response. It is also evident that the lowest plateau force measured at the deformation of 30.4% is reached as the internal pore walls come into contact at the deformation of 30%. The contact of internal pore walls prevents further pore deformation in the local region, increasing the region stiffness [52]. The sample continues to deform in stiffer regions. Therefore, the recorded force also increases. The same conclusions are valid for APM foam samples 2 and 3 which indicate the general deformation behaviour of the spherical APM foam samples.

The deformation level of the shear plane appearance and the deformation level of the first force peak can also be predicted from the data of the maximum filled radius. The maximum filled radius of APM foam sample 1 starts to increase at the deformation of 22%, which coincides with the appearance of the shear plane at the deformation of 20%. Up to the point of the shear plane appearance, the sample mostly deforms parallel to the loading direction as there is enough internal pore space for the buckling and bending of the pore walls and, therefore, the maximum filled radius remains the same. As shearing deformation appears and parts of the sample start to move in the transversal direction, the maximum filled radius increases. Thus, an increase in the maximum filled radius points to the appearance of the shear deformation. Analysing the results for samples 2 and 3 gives the same conclusions.

Analysing the rate of the change in the normalised filled volume and the average sample porosity gives similar results. For APM foam sample 1, a local minimum in the volume change was recorded at a deformation of 22% and a local minimum in the porosity change was recorded at a deformation of 20%, which coincides with the appearance of the shear plane. Up to the appearance of the shear plane, the outer volume of the sample is reduced. After initiation of the transversal shear deformation, the loading is not only converted into compression of the sample, but also in its transversal deformation and the rate of volume change is reduced. Since the average sample porosity is computed using the filled volume, this effect is also observable in the values of the average porosity. The results of samples 2 and 3 confirm these findings.

## 5. Conclusions

Observation of the compressive behaviour of APM foam samples using two different methods, micro-computed tomography coupled with in-situ compressive testing, enabled new insights into the deformation behaviour of the APM foam samples to be determined. The most important findings comparing the results of both methods are as follows:

- The analysis of the maximum filled radius of all three APM foam samples showed that the maximum filled radius remains almost constant in the first stages of the compression. Up to the deformation of 20% the maximum filled radius changes for less than 1%.
- In the first stages of the sample compression, the spherical sample shape changes the characteristic compressive response of the porous material because the regions with the smallest cross-sectional area at the surface of the sphere are compressed first and so the plateau region is reached at higher deformation levels.
- The mechanical response correlated with the internal deformation of the samples. It was found that the highest stiffness of the samples is reached before initiation of the shear plane and the resulting transversal sample deformation. After this point, the stiffness of the sample reduces up to the point of the first contact between the internal pore walls which increases the sample stiffness towards the densification region.
- The geometrical analysis of the outer shape and porosity of the APM foam samples showed that the occurrence of the shear plane is also noticeable in the values of the maximum filled radius, the filled volume and the average porosity.

## Figures and Tables

**Table 1:** Average force and deformation response at the characteristic points of the deformation response of the APM samples with standard deviations in the force and deformation at both characteristic points.

Characteristic Point	Force [N]	Deformation [%]
Local load maximum	480.1 ± 46.0	24.3 ± 6.2
Plateau region minimum	355.3 ± 62.5	34.2 ± 4.6

## Data Availability

The data supporting the findings of this study are available from the corresponding author D.K. on request.

## References

[1] Borovinšek M., Vesenjak M., Higa Y., Shimojima K., Ren Z. (2019). Characterization of geometrical changes of spherical Advanced Pore Morphology (APM) foam elements during compressive deformation. Materials.

[2] Vesenjak M., Borovinšek M., Fiedler T., Higa Y., Ren Z. (2013). Structural characterisation of advanced pore morphology (APM) foam elements. Mater. Lett..

[3] Sulong M.A., Vesenjak M., Belova I.V., Murch G.E., Fiedler T. (2014). Compressive properties of Advanced Pore Morphology (APM) foam elements. Mater. Sci. Eng. A.

[4] Stöbener K., Baumeister J., Rausch G., Busse M. (2007). Advanced Pore Morphology (APM) Metal Foams. High Temp. Mater. Process..

[5] Stöbener K. (2007). Advanced Pore Morphology (APM)—Aluminiumschaum.

[6] Stöbener K., Lehmhus D., Avalle M., Peroni L., Busse M. (2008). Aluminum foam-polymer hybrid structures (APM aluminum foam) in compression testing. Int. J. Solids Struct..

[7] Baumeister J., Monno M., Goletti M., Mussi V., Weise J. (2012). Dynamic Behavior of Hybrid APM (Advanced Pore Morphology Foam) and Aluminum Foam Filled Structures. Metals.

[8] Kovačič A., Novak N., Vesenjak M., Dobnik Dubrovski P., Ren Z. (2018). Geometrical and mechanical properties of polyamide PA 12 bonds in composite advanced pore morphology (APM) foam structures. Arch. Civ. Mech. Eng..

[9] Weise J., Queiroz Barbosa A.F., Yezerska O., Lehmhus D., Baumeister J. (2017). Mechanical Behavior of Particulate Aluminium-Epoxy Hybrid Foams Based on Cold-Setting Polymers. Adv. Eng. Mater..

[10] Lehmhus D., Weise J., Baumeister J. (2017). Cellular Metals—From Aluminium Foams to Iron/Steel Matrix Syntactic Foams.

[11] Vesenjak M., Gacnik F., Krstulovic-Opara L., Ren Z. (2011). Behavior of composite advanced pore morphology foam. J. Compos. Mater..

[12] Vesenjak M., Gačnik F., Krstulović-Opara L., Ren Z. (2015). Mechanical Properties of Advanced Pore Morphology Foam Elements. Mech. Adv. Mater. Struct..

[13] Hohe J., Hardenacke V., Fascio V., Girard Y., Baumeister J., Stöbener K., Weise J., Lehmhus D., Pattofatto S., Zeng H. (2012). Numerical and experimental design of graded cellular sandwich cores for multi-functional aerospace applications. Mater. Des..

[14] Lehmhus D., Vesenjak M., de Schampheleire S., Fiedler T. (2017). From stochastic foam to designed structure: Balancing cost and performance of cellular metals. Materials.

[15] Fiedler T., Sulong M.A., Vesenjak M., Higa Y., Belova I.V., Öchsner A., Murch G.E. (2014). Determination of the thermal conductivity of periodic APM foam models. Int. J. Heat Mass Transf..

[16] Šleichrt J., Fíla T., Koudelka P., Adorna M., Falta J., Zlámal P., Glinz J., Neuhäuserová M., Doktor T., Mauko A. (2021). Dynamic penetration of cellular solids: Experimental investigation using Hopkinson bar and computed tomography. Mater. Sci. Eng. A.

[17] Duarte I., Vesenjak M., Krstulović-Opara L., Ren Z. (2015). Compressive performance evaluation of APM (Advanced Pore Morphology) foam filled tubes. Compos. Struct..

[18] Vesenjak M., Duarte I., Baumeister J., Göhler H., Krstulović-Opara L., Ren Z. (2020). Bending performance evaluation of aluminium alloy tubes filled with different cellular metal cores. Compos. Struct..

[19] Duarte I., Krstulović-Opara L., Dias-de-Oliveira J., Vesenjak M. (2019). Axial crush performance of polymer-aluminium alloy hybrid foam filled tubes. Thin-Walled Struct..

[20] Lehmhus D., Baumeister J., Stutz L., Schneider E., Stöbener K., Avalle M., Peroni L., Peroni M. (2010). Mechanical Characterization of Particulate Aluminum Foams—Strain-Rate, Density and Matrix Alloy versus Adhesive Effects. Adv. Eng. Mater..

[21] Ulbin M., Borovinšek M., Higa Y., Shimojima K., Vesenjak M., Ren Z. (2014). Internal structure characterisation of AlSi7 and AlSi10 advanced pore morphology (APM) foam elements. Mater. Lett..

[22] Vásárhelyi L., Kónya Z., Kukovecz Á., Vajtai R. (2020). Microcomputed tomography–based characterisation of advanced materials: A review. Mater. Today Adv..

[23] Fernández M.P., Kao A.P., Witte F., Arora H., Tozzi G. (2020). Low-cycle full-field residual strains in cortical bone and their influence on tissue fracture evaluated via in situ stepwise and continuous X-ray computed tomography. J. Biomech..

[24] Vavrik D., Benes P., Fila T., Koudelka P., Kumpova I., Kytyr D., Vopalensky M., Vavro M., Vavro L. (2021). Local fracture toughness testing of sandstone based on X-ray tomographic reconstruction. Int. J. Rock Mech. Min. Sci..

[25] Elkhoury J.E., Shankar R., Ramakrishnan T.S. (2019). Resolution and Limitations of X-Ray Micro-CT with Applications to Sandstones and Limestones. Transp. Porous Media.

[26] Wan T., Liu Y., Zhou C., Chen X., Li Y. (2021). Fabrication, properties, and applications of open-cell aluminum foams: A review. J. Mater. Sci. Technol..

[27] Nickerson S., Shu Y., Zhong D., Könke C., Tandia A. (2019). Permeability of porous ceramics by X-ray CT image analysis. Acta Mater..

[28] Senck S., Glinz J., Happl M., Scheerer M., Reiter T., Kastner J. (2021). Quantification of surface-near porosity in additively manufactured aluminum brackets using x-ray microcomputed tomography. Proceedings of the AIAA Scitech 2021 Forum.

[29] Parveez B., Jamal N.A., Maleque A., Yusof F., Jamadon N.H., Adzila S. (2021). Review on advances in porous Al composites and the possible way forward. J. Mater. Res. Technol..

[30] Afolabi L.O., Ariff Z.M., Hashim S.F.S., Alomayri T., Mahzan S., Kamarudin K.A., Muhammad I.D. (2020). Syntactic foams formulations, production techniques, and industry applications: A review. J. Mater. Res. Technol..

[31] Li Y., Jahr H., Zhou J., Zadpoor A.A. (2020). Additively manufactured biodegradable porous metals. Acta Biomater..

[32] Benedetti M., du Plessis A., Ritchie R.O., Dallago M., Razavi S.M.J., Berto F. (2021). Architected cellular materials: A review on their mechanical properties towards fatigue-tolerant design and fabrication. Mater. Sci. Eng. R Rep..

[33] Hassanli F., Paydar M.H. (2021). Improvement in energy absorption properties of aluminum foams by designing pore-density distribution. J. Mater. Res. Technol..

[34] Li W., Xu K., Li H., Jia H., Liu X., Xie J. (2017). Energy Absorption and Deformation Mechanism of Lotus-type Porous Coppers in Perpendicular Direction. J. Mater. Sci. Technol..

[35] Hao M., Wei C., Liu X., Ge Y., Cai J. (2021). Quantitative evaluation on mechanical characterisation of Ti6Al4V porous scaffold designed based on Weaire-Phelan structure via experimental and numerical analysis methods. J. Alloys Compd..

[36] Wu F., Liu T., Xiao X., Zhang Z., Hou J. (2019). Static and dynamic crushing of novel porous crochet-sintered metal and its filled composite tube. Compos. Struct..

[37] Hajizadeh M., Yazdani M., Vesali S., Khodarahmi H., Mirzababaie Mostofi T. (2021). An experimental investigation into the quasi-static compression behavior of open-cell aluminum foams focusing on controlling the space holder particle size. J. Manuf. Process..

[38] Forna-Kreutzer J.P., Ell J., Barnard H., Pirzada T.J., Ritchie R.O., Liu D. (2021). Full-field characterisation of oxide-oxide ceramic-matrix composites using X-ray computed micro-tomography and digital volume correlation under load at high temperatures. Mater. Des..

[39] Fernández M.P., Kao A.P., Bonithon R., Howells D., Bodey A.J., Wanelik K., Witte F., Johnston R., Arora H., Tozzi G. (2021). Time-resolved in situ synchrotron-microCT: 4D deformation of bone and bone analogues using digital volume correlation. Acta Biomater..

[40] Hangai Y., Kawato D., Ohashi M., Ando M., Ogura T., Morisada Y., Fujii H., Kamakoshi Y., Mitsugi H., Amagai K. (2021). X-ray Radiography Inspection of Pores of Thin Aluminum Foam during Press Forming Immediately after Foaming. Metals.

[41] Heitor D., Duarte I., Dias-De-oliveira J. (2021). Aluminium alloy foam modelling and prediction of elastic properties using x-ray microcomputed tomography. Metals.

[42] Marone F., Studer A., Billich H., Sala L., Stampanoni M. (2017). Towards on-the-fly data post-processing for real-time tomographic imaging at TOMCAT. Adv. Struct. Chem. Imaging.

[43] Kytýř D., Zlámal P., Koudelka P., Fíla T., Krčmářová N., Kumpová I., Vavřík D., Gantar A., Novak S. (2017). Deformation analysis of gellan-gum based bone scaffold using on-the-fly tomography. Mater. Des..

[44] Costanza G., Giudice F., Sili A., Tata M.E. (2021). Correlation Modeling between Morphology and Compression Behavior of Closed-Cell Al Foams Based on X-ray Computed Tomography Observations. Metals.

[45] Ghazi A., Berke P., Tiago C., Massart T.J. (2020). Computed tomography based modelling of the behaviour of closed cell metallic foams using a shell approximation. Mater. Des..

[46] Jiroušek O., Doktor T., Kytýř D., Zlámal P., Fíla T., Koudelka P., Jandejsek I., Vavřík D. (2013). X-ray and finite element analysis of deformation response of closed-cell metal foam subjected to compressive loading. J. Instrum..

[47] Fíla T., Šleichrt J., Kytýř D., Kumpová I., Vopálenský M., Zlámal P., Rada V., Vavřík D., Koudelka P., Senck S. (2018). Deformation analysis of the spongious sample in simulated physiological conditions based on in-situ compression, 4D computed tomography and fast readout detector. J. Instrum..

[48] Rada V., Fíla T., Zlámal P., Kytýř D., Koudelka P. (2018). Multi-channel control system for in-situ laboratory loading devices. Proceedings of the 16th Youth Symposium on Experimental Solid Mechanics.

[49] Miao H., Zhao H.J., Gao F., Gong S.R. Implementation of FDK reconstruction algorithm in cone-beam CT based on the 3D Shepp-Logan Model. Proceedings of the BMEI 2009: 2nd International Conference on Biomedical Engineering and Informatics.

[50] Wang X., Zhou Y., Li J., Li H. (2021). Uniaxial compression mechanical properties of foam nickel/iron-epoxy interpenetrating phase composites. Materials.

[51] Gibson L.J., Ashby M.F. (1997). Cellular Solids: Structure and Properties.

[52] Byakova A., Gnyloskurenko S., Vlasov A., Semenov N., Yevych Y., Zatsarna O., Danilyuk V. (2019). Effect of Cell Wall Ductility and Toughness on Compressive Response and Strain Rate Sensitivity of Aluminium Foam. Adv. Mater. Sci. Eng..

